# Evaluation of Transcriptomic Responses in Livers of Mice Exposed to the Short-Chain PFAS Compound HFPO-DA

**DOI:** 10.3389/ftox.2022.937168

**Published:** 2022-06-27

**Authors:** Melissa M. Heintz, Grace A. Chappell, Chad M. Thompson, Laurie C. Haws

**Affiliations:** ^1^ ToxStrategies, Inc, Asheville, NC, United States; ^2^ ToxStrategies, Inc, Katy, TX, United States; ^3^ ToxStrategies, Inc, Austin, TX, United States

**Keywords:** dose-response, GenX, HFPO-DA, per and polyfluoroalkyl substances (PFAS), peroxisome proliferator-activated receptor alpha (PPARα), transcriptomics

## Abstract

HFPO-DA (ammonium 2,3,3,3-tetrafluoro-2-(heptafluoropropoxy)-propanoate; CASRN 62037-80-3) is a component of the GenX technology platform used as a polymerization aid in the manufacture of some types of fluoropolymers. The liver is the primary target of toxicity for HFPO-DA in rodents and previous examination of hepatic transcriptomic responses in mice following oral exposure to HFPO-DA for 90 days showed induction of peroxisome proliferator-activated receptor signaling pathways, predominantly by PPARα, as well as increased gene expression of both peroxisomal and mitochondrial fatty acid metabolism. To further investigate the mechanism of liver toxicity, transcriptomic analysis was conducted on liver tissue from mice orally exposed to 0, 0.1, 0.5 or 5 mg/kg-bw/day HFPO-DA in a reproduction/developmental toxicity study. Hepatic gene expression changes demonstrated activation of the PPARα signaling pathway. Peroxisomal and mitochondrial fatty acid β-oxidation gene sets were enriched at lower HFPO-DA concentrations, and complement cascade, cell cycle and apoptosis related gene sets were enriched at higher HFPO-DA concentrations. These results support the reported histopathological findings in livers of mice from this study and indicate that the effects of HFPO-DA are mediated through rodent-specific PPARα signaling mechanisms regardless of reproductive status in mice.

## Introduction

HFPO-DA (ammonium 2,3,3,3-tetrafluoro-2-(heptafluoropropoxy)-propanoate; CASRN 62037-80-3) is used as a polymerization aid in the manufacture of some types of fluorinated polymers. These fluoropolymers are then used in multiple applications such as semiconductor fluid handling, high-purity chemical processing, aerospace and telecommunications cabling, renewable hydrogen production, and lithium-ion batteries in transportation.^1^,^2^ Although HFPO-DA is sometimes referred to broadly as a perfluorooctanoate acid (PFOA) replacement, different fluoropolymer manufacturers have developed their own replacement polymerization aid technologies to replace PFOA (Wang et al., 2013). In contrast to the myriad of historical uses of PFOA (Prevedouros et al., 2006), HFPO-DA is not used or found in firefighting foam, carpets, textiles, paper or other industrial consumer products that have been associated with PFOA in the past. In contrast to long-chain per- and poly fluorinated alkyl substances (PFAS) such as PFOA, studies have shown that HFPO-DA is rapidly eliminated and does not bioaccumulate in tissues (Gannon et al., 2016).

HFPO-DA has been evaluated in both short- and long-term toxicity studies, with results demonstrating that the liver is the primary target of toxicity in rodents following oral exposure (Thompson et al., 2019; USEPA, 2021). Previous transcriptomic analysis of livers collected from a mouse 90-days subchronic oral gavage study of HFPO-DA showed up-regulation of peroxisome proliferator-activated receptor (PPAR) signaling pathways (i.e., predominantly PPARα) and both peroxisomal and mitochondrial fatty acid metabolism at the gene-expression level (Chappell et al., 2020). To further elucidate the mechanism of liver toxicity for HFPO-DA in mice presented in Chappell et al. (2020) and examine whether reproductive status alters molecular responses, we performed whole transcriptomic analysis on mouse livers from an Organisation for Economic Co-operation and Development (OECD) 421 guideline reproduction/developmental toxicity study of HFPO-DA (Dupont Chem, 2010). Understanding the transcriptomic responses in this specific study is important as the United States Environmental Protection Agency (USEPA) has based their chronic (0.000003 mg/kg/day)^3^ and subchronic (0.00003 mg/kg/day) reference dose (RfD) on the observed liver effects in this study (USEPA, 2021). The transcriptomic results presented herein provide additional support for determining the mechanisms underlying the liver effects observed in HFPO-DA-exposed in mice, including insight into which receptor-mediated signaling pathways play a significant role.

## Methods

### Animal Exposure and Tissue Preparation

The reproduction/developmental toxicity of HFPO-DA was evaluated in a GLP OECD 421 test guideline-compliant oral gavage study in male and female Crl:CD1(ICR) mice as described previously (Dupont Chem, 2010). The technical report is publicly available in USEPA’s Health & Environmental Research Online (HERO) database (HERO ID: 4222148). Briefly, male and female mice were administered deionized water (vehicle control) or HFPO-DA (ammonium salt) in deionized water at 0.1, 0.5 or 5 mg/kg (n = 25 per dose group per sex) by oral gavage once daily for a total of 84–85 or 53–65 doses, respectively. Male mice (F_0_, approximately 6 weeks old) were dosed 70 days prior to mating through completion of mating period (14 days). Female mice (F_0_, approximately 11 weeks old) were dosed 14 days prior to mating through lactation day 20 or 21. Additional study details including test substance source and purity, analytical chemistry analyses, animal husbandry, and randomization procedure are described in the Dupont Chem (2010) study report publicly available in USEPA’s HERO database (HERO ID: 4222148). Following the last treatment, mice were euthanized by CO_2_ anesthesia and exsanguination. Livers from adult mice (F_0_) were fixed in 10% neutral-buffered formalin, embedded in paraffin (FFPE), and sections approximately 4–6 µm in thickness were mounted to glass slides.

### RNA Sequencing

Mounted, unstained FFPE liver sections from HFPO-DA-exposed male and female mice (F_0_, n = 5 per dose group per sex, total of 40 samples) were scraped from the slides and processed according to the TempO-Seq^®^ protocol by BioSypder Technologies (Carlsbad, California), as previously described (Yeakley et al., 2017). Resultant DNA libraries were sequenced using a HiSeq 2500 Ultra-High-Throughput Sequencing System (Illumina, San Diego, California).

### Data Processing and Analysis

Sequencing data were analyzed using packages in the R software environment, version 4.0.2 (cran.r-project.org/). The number of sequenced reads per TempO-Seq probe were extracted from FASTQ files generated from the sequencing experiment, with each probe representing a gene-specific sequence. Samples were excluded from the comparative analysis if either or both of the following exclusion criteria were met: overall sequencing depth (total reads across all probes) lower than two standard deviations below the mean sequencing depth across all samples; total number of sequenced probes lower than two standard deviations below the mean number of probes sequenced per sample. Count data from all samples that were not excluded were used for further comparative analyses.

### Identification of Differentially Expressed Genes Across Treatment Groups

The DESeq2 R package (v1.28.1) (Love et al., 2014) was used to normalize the data such that sample-to-sample variation in sequencing depth was considered. Statistical methods within DESeq2 were used to calculate fold-change and identify differentially expressed genes (DEGs) associated with HFPO-DA by conducting statistical comparisons between treatment groups and controls of the same sex. Differentially expressed probes (DEPs) were defined as those with a false discovery rate (FDR) < 10%, based on *p* values adjusted for multiple testing using the Benjamini and Hochberg (BH) procedure (Love et al., 2014); DEGs were identified from respective DEPs, as some genes (but not all) are represented by multiple probes in the TempO-Seq assay.

### Identification of Pathway-Level Alterations Across Concentrations

Biological pathways associated with transcriptomic response profiles were identified by gene set enrichment analysis. For genes for which multiple probes were used to measure expression, the probe with the highest sequencing count across all samples was used in the pathway analyses. Mouse gene identifiers were converted into human identifiers using the R package biomaRt (v2.38.0) based on the Ensembl genome database (http://uswest.ensembl.org/index.html). Gene expression data were then queried for enrichment of gene sets within the canonical pathway (CP) subcollection (c2.cp.v7.5.1) available through the Molecular Signatures Database (MSigDB: http://software.broadinstitute.org/gsea/msigdb/index.jsp), which includes gene sets from several pathway databases.

Enrichment of gene sets and pathways was evaluated by two methods. The first method follows the analysis employed by the gene set enrichment analysis (GSEA) platform made available by the Broad Institute (http://software.broadinstitute.org/gsea/index.jsp), and the second method employed a hypergeometric test for over-representation. The GSEA method (Subramanian et al., 2005) determines whether sets of genes (e.g., the constituents of a molecular signaling pathway) are significantly concordant between various defined groups (in the case presented herein, different dose levels by sex) based on a ranking metric (in this case, the statistical measure of differences in expression between treated and control mice, using the Wald statistic as determined with DESeq2). The GSEA statistical method was applied within the Platform for Integrative Analysis of Omics data (PIANO) R package (v1.22.0) (Varemo et al., 2013). Gene set enrichment significance was calculated using permutation-based nominal *p* values based on weighted Kolmogorov-Smirnov test enrichment scores and adjusted for multiple hypothesis testing by calculating FDRs using the BH method (Subramanian et al., 2005). For the hypergeometric test, only DEGs for each treatment group (i.e., an FDR of <10% as described above) were tested for overrepresentation among the gene sets in the CP subcollection using the Fisher combined probability test function within the PIANO package. For both methods, gene sets with an FDR <10% were considered significantly enriched.

### Canonical Pathway and Upstream Regulator Prediction Analyses

Ingenuity Pathway Analysis (IPA, v. 01-20-04; Qiagen Bioinformatics, Redwood City, California) was used to identify canonical pathway associations to HFPO-DA treatment and inferred upstream regulators associated with DEGs. Fold change and statistical values determined by DESeq2 for all DEGs (i.e., DEGs with FDR <10%) were used to conduct the analyses.

### Benchmark Dose Analysis

Dose–response modeling was conducted using the BMDExpress software (v2.3) (Phillips et al., 2019). Normalized expression data for all samples as generated using DESeq2 were loaded into BMDExpress without transformation, using probe IDs from the TempO-Seq experiment as gene identifiers. A Williams trend test (with *p* value cutoff = 0.05) was used to identify genes altered by exposure to HFPO-DA. No fold-change filters or correction for multiple tests were applied. Benchmark dose (BMD) analysis was conducted using the following models: linear, power, hill, 2° and 3° polynomial, and exponential models 2–5. The models were run assuming constant variance and a benchmark response (BMR) of 1 standard deviation. Dose-responsive genes with a best BMD >10-fold below the lowest dose (0.1 mg/kg) or a best BMD > the highest dose (5 mg/kg) were removed. Functional classification was conducted using the gene set collections available within the BMDExpress software (Reactome gene sets), based on significantly dose-responsive genes (i.e., genes with a winning model fit *p* value ≥0.1), and removing genes according to the default parameters as follows: genes with BMD/BMDL >20, BMDU/BMD >20, and BMDU/BMDL >40. No filters for minimum or maximum number of genes per gene set were applied. Benchmark doses for the gene sets were also calculated.

## Results

Detailed results of the toxicological endpoints collected during the reproduction/developmental toxicity study are not reported herein but are provided in the original technical report (Dupont Chem, 2010) which, as outlined above, is publicly available in USEPA’s HERO database (HERO ID: 4222148). Briefly, no adverse effects on reproduction or postnatal survival were observed. Liver effects including increased liver weight, hepatocellular hypertrophy, and other histopathological changes were observed in male and female (F_0_) adults administered 0.5 or 5 mg/kg HFPO-DA. Decreased pup body weight was observed in both sexes in the highest dose group (5 mg/kg).

The expression levels of 21,448 mouse genes as measured by 30,146 probes were reported from the TempO-Seq assay using liver samples from the reproduction/developmental toxicity study of HFPO-DA at doses of 0.1, 0.5, and 5 mg/kg bw/day (Supplementary Table S1). Three samples were removed from the analysis (all from different dose/sex groups) because the sequencing data quality did not meet criteria described in the Methods section (Supplementary File S1).

### Transcriptomic Changes Associated With HFPO-DA

**FIGURE 1:**
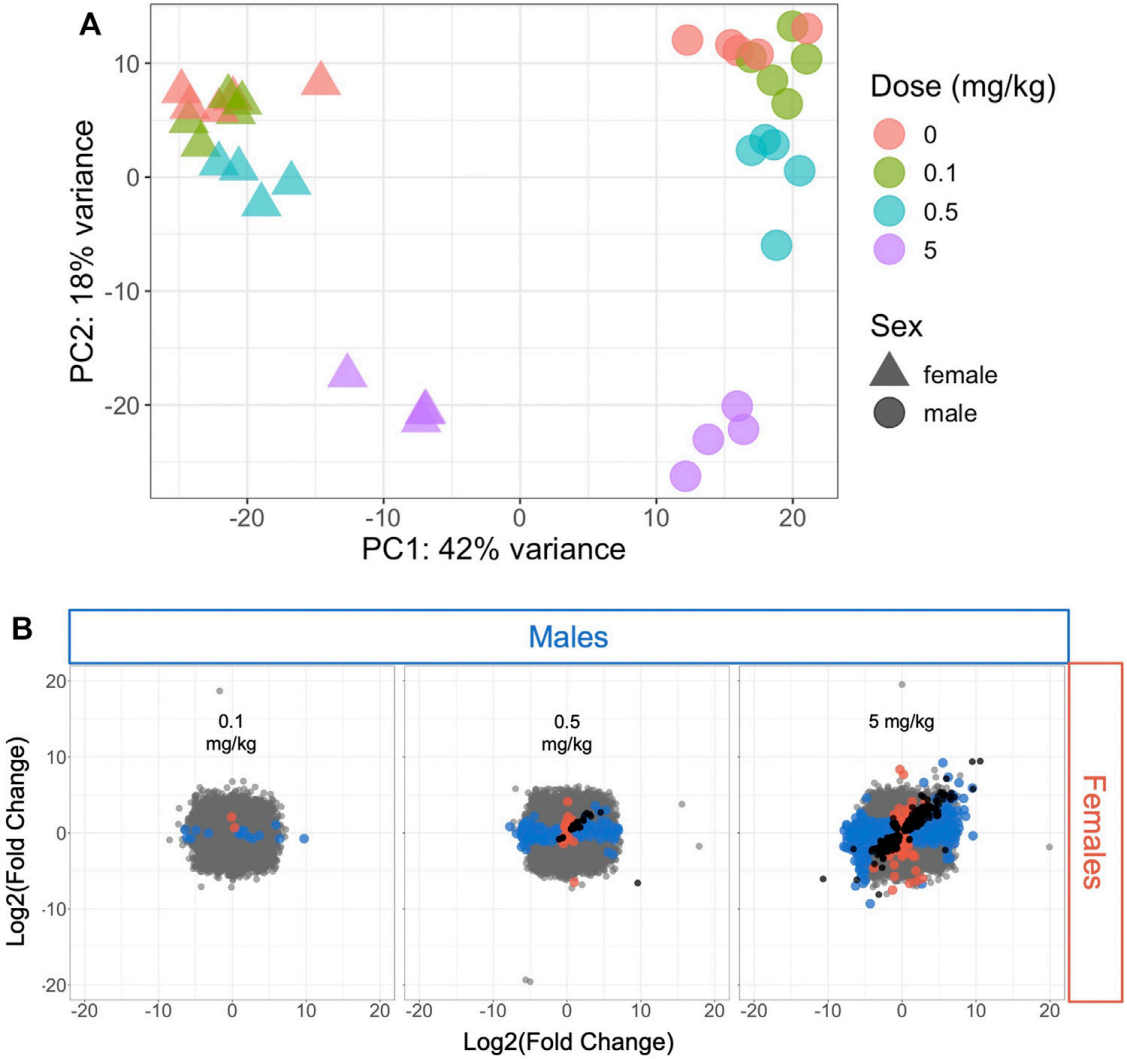
Hepatic transcriptomic profile comparison of all samples or significant differentially expressed genes (DEGs) in male and female mice **(A)** Principal components analysis plot of all samples included in the analysis. Each shape represents a sample, with sex and dose level indicated by triangles (females) or circles (males) and color **(B)** Comparison of DEGs associated with each HFPO-DA exposure level between male and female mice. The fold change (exposed/control) in hepatic mRNA level for all genes in male and female mice is plotted on the x- and *y*-axes, respectively. Significant DEGs (FDR <10%, relative to controls) are shown by color-coded points according to sex (blue color for males, salmon color for females). Black points represent significant DEGs shared between both sexes at a given dose level, whereas gray points represent non-significant DEGs.

The variance in transcriptomic profiles across the dataset was visualized using principal components analysis (PCA) (Figure 1A). The samples varied the most by sex (PC1) followed by dose (PC2), with the highest dose (5 mg/kg) having the greatest difference from the control samples. The mid- and low-dose (0.5 and 0.1 mg/kg, respectively) samples showed less variation from the control samples and from each other (Figure 1A). This pattern was consistent with the number of significantly differentially expressed genes (DEGs) at each dose level compared to controls in male and female mice (Figure 1B; Supplementary Table S1): very few genes were altered by 0.1 mg/kg HFPO-DA compared to controls (19 and 3 DEGs in male and female mice, respectively), increasing to 293 and 66 DEGs in male and female mice, respectively, at 0.5 mg/kg, and 2,460 and 1,065 DEGs in male and female mice, respectively at 5 mg/kg HFPO-DA.

**FIGURE 2:**
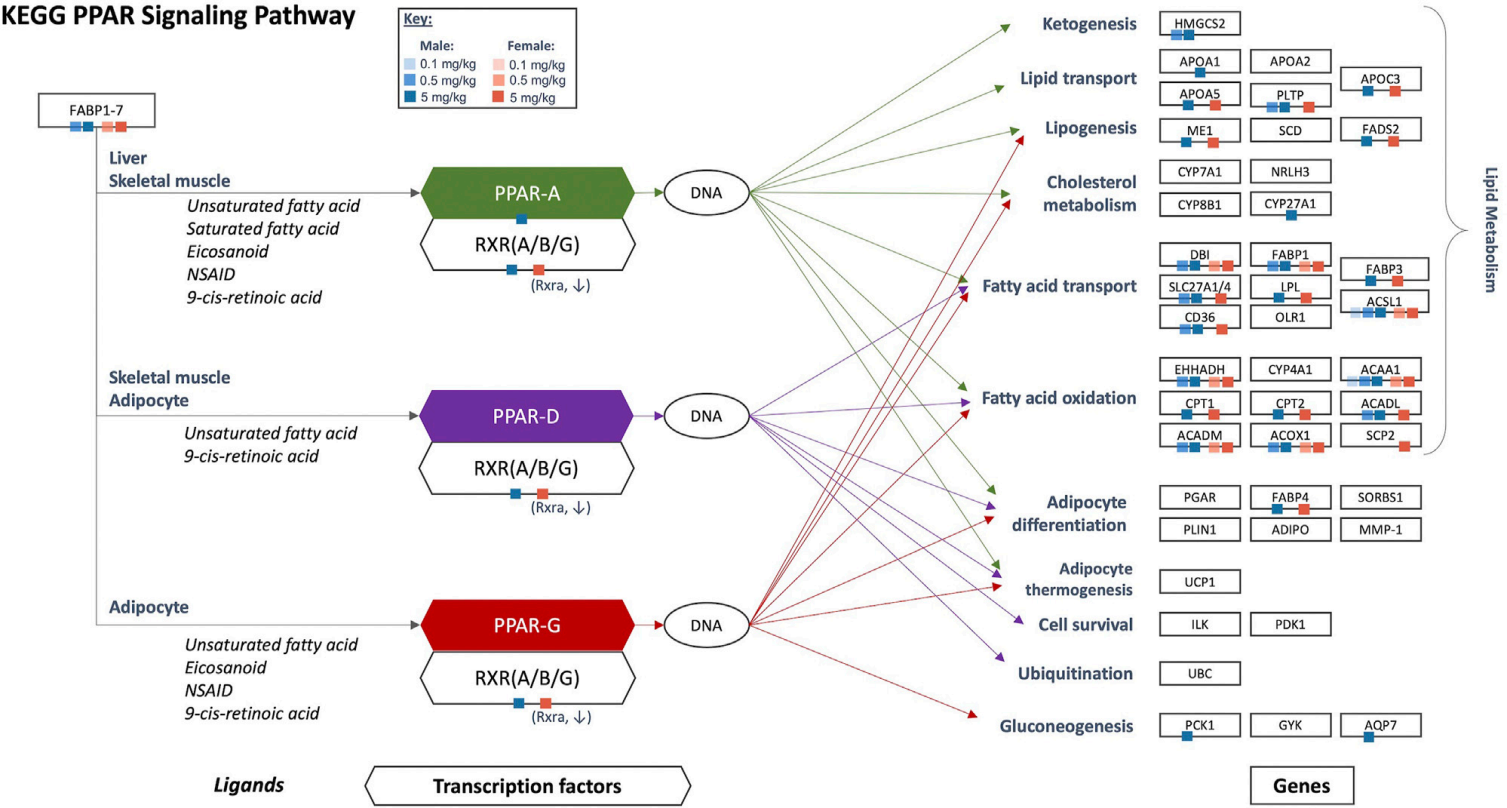
Significant DEGs that are a part of the KEGG PPAR signaling network in HFPO-DA-exposed male and female mice. Ligands, transcription factors, and genes, as related to PPAR α/γ/δ signaling, are shown according to the KEGG database. Individual PPAR signaling genes that are significantly differentially expressed in the present study are notated by color-coded squares by sex and concentration. Arrows show the target genes corresponding to each PPAR isoform: green = PPARα, purple = PPARδ, red = PPARγ. Figure adapted from Chappell et al. (2020). KEGG, Kyoto Encyclopedia of Genes and Genomes; PPAR, peroxisome proliferator-activated receptor.

Results of gene set enrichment analyses from the GSEA and hypergeometric test methods are consistent with the results presented in Chappell et al. (2020). Specifically, the most significantly enriched gene sets at 0.5 and 5 mg/kg HFPO-DA for both sexes include up-regulated fatty acid metabolism, PPAR signaling (i.e., “KEGG PPAR Signaling Pathway” and “WP PPAR Signaling”), and mitochondrial and peroxisomal fatty acid β-oxidation, as well as down-regulation of complement and coagulation cascades (Supplementary Tables S2, S3). While general PPAR signaling gene sets were among the most significantly upregulated, the specificity for PPARα activation by HFPO-DA is demonstrated by the DEGs that are specific to the α isoform and a general lack of changes in expression levels of most of the genes specific to other PPAR isoforms (Figure 2). Gene sets specific to the PPAR alpha (PPARα) subtype are significantly enriched in both male and female mice at 0.5 and 5 mg/kg HFPO-DA (Table 1), while PPAR gamma (PPARγ) signaling gene sets are not significantly enriched in either sex at any dose (Table 1). Gene sets specific to PPAR delta (PPARδ) are not currently available within the canonical pathway subcollection used for gene set enrichment analyses herein.

**TABLE 1:** Comparison of enriched gene sets for general peroxisome/PPAR signaling and PPARα-versus PPARγ-specific gene sets in HFPO-DA-exposed mice.

Groups of canonical pathways	Gene set	Sex	HFPO-DA Mg/Kg-Bw/day	GSEA pre-ranked method		Hypergeometric method	
				Adjusted *p*-value*	Overall direction	Adjuste *p*-value*	Overall direction
**General PPAR/Peroxisomal Signaling**	**KEGG Peroxisome**	Female	0.1	0.67207	NS	1	NS
			0.5	**0**	Up	**6.8911E-09**	Up
			5	**0**	Up	**4.6811E-14**	Up
		Male	0.1	**0.025315**	Up	1	NS
			0.5	**0**	Up	**1.8276E-09**	Up
			5	**0**	Up	**4.9931E-12**	Up
	**KEGG PPAR Signaling**	Female	0.1	0.59594	NS	1	NS
			0.5	**0**	Up	**3.1184E-09**	Up
			5	**0**	Up	**1.6363E-17**	Up
		Male	0.1	**0.046292**	Up	1	NS
			0.5	**0**	Up	**4.4076E-14**	Up
			5	**0**	Up	**1.8259E-12**	Up
	**REACTOME Peroxisomal Lipid Metabolism**	Female	0.1	0.73987	NS	1	NS
			0.5	**0**	Up	**2.388E-08**	Up
			5	**0**	Up	**3.9408E-09**	Up
		Male	0.1	0.085653	NS	1	NS
			0.5	**0**	Up	**4.0131E-08**	Up
			5	**0**	Up	**3.9031E-06**	Up
	**WP PPAR Signaling**	Female	0.1	0.61457	NS	1	NS
			0.5	**0**	Up	**5.542E-08**	Up
			5	**0**	Up	**1.0497E-16**	Up
		Male	0.1	0.079179	NS	1	NS
			0.5	**0**	Up	**7.0315E-13**	Up
			5	**0**	Up	**5.5036E-12**	Up
**PPARα signaling**	**BIOCARTA PPARα Pathway**	Female	0.1	0.57884	NS	1	NS
			0.5	**0.03138**	Up	**0.00078767**	Up
			5	**0.0059801**	Up	**0.00847825**	Up
		Male	0.1	0.74593	NS	1	NS
			0.5	**0.0081228**	Up	**0.00769699**	Up
			5	**0.052564**	Up	0.4160496	NS
	**REACTOME Regulation of Lipid Metabolism by PPARα**	Female	0.1	0.58391	NS	1	NS
			0.5	0.077991	NS	**0.01161897**	Up
			5	**0.020328**	Up	**0.00573576**	Up
		Male	0.1	0.67953	NS	1	NS
			0.5	**0.0066388**	Up	**2.3197E-05**	Up
			5	**0.044658**	Up	**0.00826333**	Up
	**WP PPARα Pathway**	Female	0.1	0.50683	NS	1	NS
			0.5	**0**	Up	**7.902E-07**	Up
			5	**0.00058828**	Up	**1.8792E-08**	Up
		Male	0.1	**0.023999**	Up	1	NS
			0.5	**0**	Up	**1.6442E-08**	Up
			5	**0.001163**	Up	**9.3209E-05**	Up
**PPARγ signaling**	**BIOCARTA PPARγ Pathway**	Female	0.1	0.94989	NS	1	NS
			0.5	0.78706	NS	1	NS
			5	0.93323	NS	1	NS
		Male	0.1	0.85089	NS	1	NS
			0.5	0.89257	NS	1	NS
			5	0.9111	NS	1	NS
	**WP HIF1α and PPARγ Regulation of Glycolysis**	Female	0.1	0.56814	NS	1	NS
			0.5	0.97801	NS	1	NS
			5	0.060278	NS	1	NS
		Male	0.1	0.98959	NS	1	NS
			0.5	0.58395	NS	1	NS
			5	0.51884	NS	0.7100514	NS

*Bold formatting indicates significant adjusted *p*-value (i.e., FDR ≤0.05); NS = Not significant (FDR >0.05).

Significantly enriched canonical pathways were also identified using QIAGEN Ingenuity Pathway Analysis (IPA) (QIAGEN Inc., https://digitalinsights.qiagen.com/IPA) (Supplementary Table S4) and were comparable to the significantly enriched gene sets determined by the GSEA and hypergeometric test methods. Additionally, in both sexes, IPA upstream analyses predicted PPARα as the most significant upstream regulator at 5 mg/kg HFPO-DA and the second most significant upstream regulator at 0.5 mg/kg HFPO-DA (Supplementary Table S5). Due to the lack of transcriptomic response in mice exposed to 0.1 mg/kg HFPO-DA (i.e., only 19 and 3 DEGs in males and females, respectively), upstream prediction analysis was not performed.

### Benchmark Dose Modeling of Gene Expression Data

**FIGURE 3:**
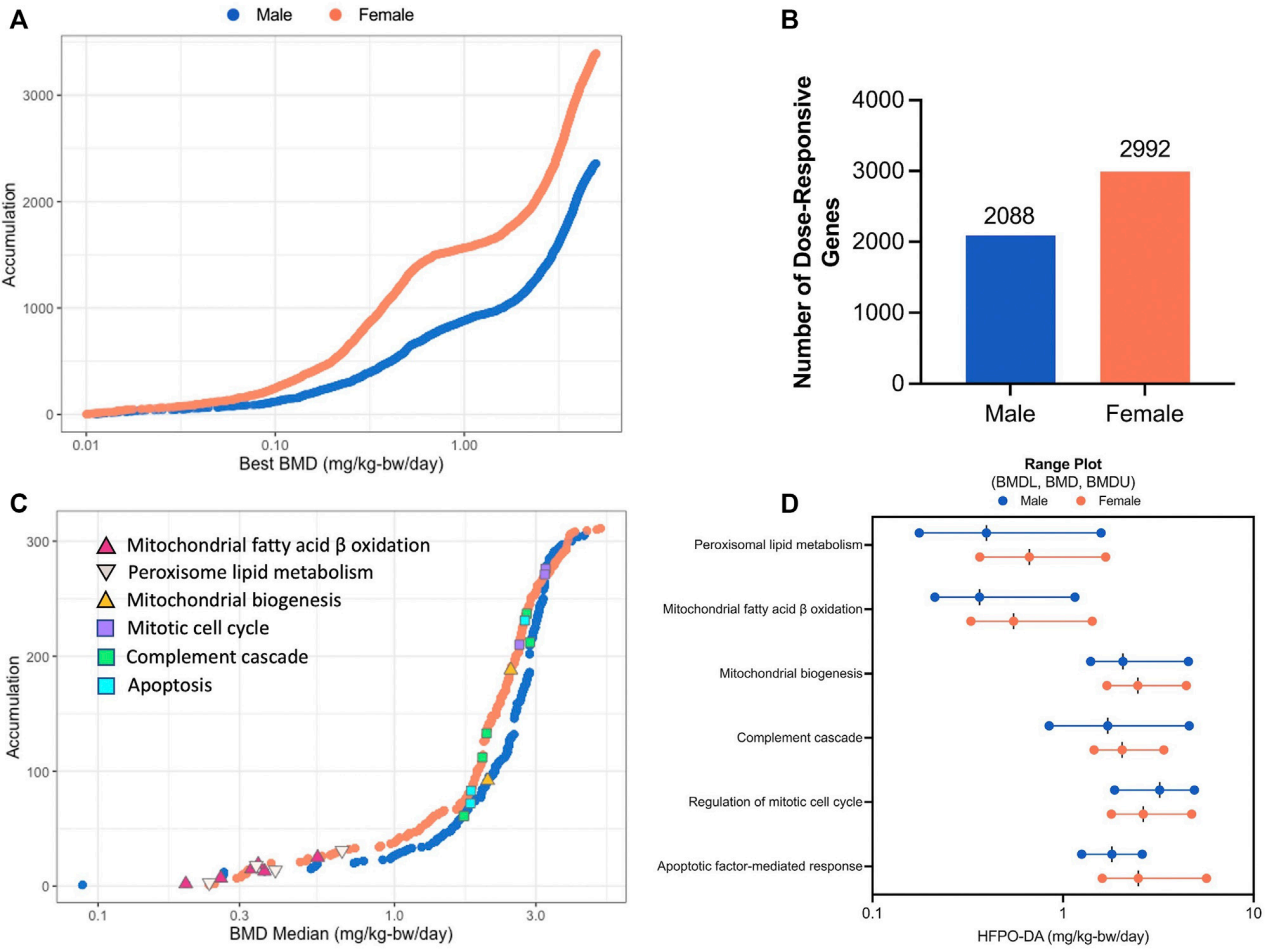
BMD analysis visualizations **(A)** Accumulation plots of modeled benchmark doses among significant dose-responsive probes in male and female mice (best fit *p*-value ≥ 0.1) **(B)** Number of significant dose-responsive genes by sex (best fit *p*-value ≥ 0.1) **(C)** Accumulation plots for significantly enriched pathways in male and female mice (Fisher’s Exact Two-Tail < 0.1) with selected gene sets annotated by color-coded triangles and squares as indicated in the inset legend **(D)** Range plots (median BMDL, median BMD, median BMDU) for the same selected gene sets depicted by triangles and squares in figure panel **(C)**.

The dose-response across all genes was modeled using BMDExpress software (v2.3) (Phillips et al., 2019). To avoid extrapolation too far below or above the range of empirical data, 86 and 123 dose-responsive genes with BMD values more than 10-fold below the lowest dose (0.1 mg/kg), in addition to, 60 and 86 dose-responsive genes with BMD values greater than the highest dose (5 mg/kg), were removed from the analysis, leaving a total of 2,088 and 2,992 significant dose-responsive genes in males and females, respectively, as determined by a winning model fit *p*-value ≥ 0.1 (Figure 3A,B; Supplementary Table S6; Supplementary File S2). Among these dose-response genes, 909 were common between sexes. Functional classification (i.e., enrichment of signaling pathways) of significant dose-responsive genes results were similar to the gene set enrichment analysis conducted on individual treatment groups (described in the previous section). Specifically, the top-most significantly enriched gene sets among dose-responsive genes were related to fatty acid metabolism in both sexes (e.g., “Fatty acid metabolism” median BMD 0.61–0.79 mg/kg-bw/day) (Supplementary Table S7). In addition, gene sets related to fatty acid metabolism in the mitochondria (median BMD 0.36–0.55 mg/kg-bw/day) or peroxisome (median BMD 0.40–0.67 mg/kg-bw/day) and complement cascade (median BMD 1.72–2.05 mg/kg-bw/day) were also significantly enriched (Fisher’s Two-Tailed test <0.1) in both sexes (Figure 3C,D). Fatty acid metabolism-associated gene sets had lower median BMDs (e.g., median BMDs 0.36–0.67 mg/kg-bw/day), whereas other significantly enriched gene sets such as complement cascade, apoptosis, mitochondrial biogenesis, and mitotic cell cycle had higher median BMDs across both sexes (e.g., median BMDs 1.72–3.23 mg/kg-bw/day) (Figure 3C). Generally, these enriched gene sets had similar median BMDs for both sexes, with males being slightly lower (Figure 3D; Supplementary Table S7). These transcriptomic results are consistent with Chappell et al. (2020), with evidence for increased peroxisomal activity in the lower dose groups (0.1 and 0.5 mg/kg HFPO-DA) and apoptosis and mitosis in the high-dose group (5 mg/kg HFPO-DA). In addition, these data are also consistent with histopathological evidence of hepatocellular hypertrophy occurring at lower concentrations than apoptosis and mitotic figures in liver sections from this study (Supplementary Table S8).

## Discussion

Findings from the whole transcriptomic analysis on mouse livers from an oral reproduction/developmental toxicity study of HFPO-DA presented herein are consistent with previous hepatic transcriptomic results from an oral 90-days subchronic study of HFPO-DA (Chappell et al., 2020). Altered expression of genes within PPARα-specific signaling and lipid metabolism pathways (e.g., peroxisomal and mitochondrial fatty acid β-oxidation) resulted in highly significant enrichment of these gene sets, demonstrating that transcriptomic responses to HFPO-DA are mediated through PPARα. In addition, PPARα was the most statistically significant predicted upstream regulator of transcriptomic responses.

The underlying mechanism of HFPO-DA appears to be the same in both sexes and not affected by reproductive status, however, consistent with Chappell et al. (2020), male mice appear to be slightly more sensitive to HFPO-DA at the transcriptomic level based on the overall higher number of DEGs and the lower median BMDs for several key signaling pathways. Peroxisomal activity was observed in all dose groups whereas increased mitosis and apoptosis only occurred in the high-dose groups. Consistent with these expression patterns, the BMDs for peroxisomal activity were lower than for mitosis and apoptosis. These gene changes are consistent with histopathological and biochemical evidence of hepatocellular hypertrophy and acyl-CoA enzyme activity beginning in the low and intermediate dose groups and mitotic and apoptotic hepatocytes in the high-dose group. Although there is clear evidence for apoptosis, cell death via necrosis using transcriptomic data is limited by a lack of known molecular signals related to the phenomenon, with the exception of regulated necrosis, also known as necroptosis (Negroni et al., 2015). As the name implies, unregulated necrosis is not controlled/initiated by receptor-mediated signaling pathways and therefore has no known transcriptomic signature. Dose-responsive gene sets for regulated necrosis and necroptotic cell death were not significantly enriched in any dose group, indicating that apoptosis and autophagy are the only forms of regulated cell death occurring in livers of HFPO-DA-exposed mice. Both autophagy and apoptosis are associated with PPARα activation in rodents, as PPARα regulates autophagy in the liver (Byun et al., 2020) and increased hepatic apoptosis often coincides with increased mitosis as part of the key events in the PPARα mode of action for rodent liver tumors (Corton et al., 2014; Thompson et al., 2019). PPARα is expressed in many species and regulates lipid metabolism across species (including humans), but the altered cell growth and survival pathways (i.e., increased mitosis and apoptosis) occur solely in rodents (Corton et al., 2018; Mcmullen et al., 2020).

In addition to corroborating results from the 90-days mouse study of HFPO-DA (Chappell et al., 2020), transcriptomic analyses presented in the current study also demonstrated that only PPAR gene sets specific to the PPARα subtype were significantly enriched, in both the mid- and high-dose groups, whereas gene sets specific to PPARγ were not significantly enriched in any dose group. The lack of PPARγ gene set enrichment in the liver is likely because PPARγ is predominantly expressed in adipose tissues (Chawla et al., 1994); in comparison, PPARα, is predominantly expressed in the liver (Corrales et al., 2018). These results provide additional *in vivo* mechanistic support in rodents that confirms *in vitro* data available for both rodents and humans demonstrating the primary activation of PPARα by HFPO-DA and little to no activation of other PPAR isoforms or nuclear receptors (Behr et al., 2020; Chappell et al., 2020; Nielsen et al., 2022). Furthermore, the heterodimer partner of PPARα, retinoid X receptor alpha (RXRα), was significantly downregulated in the livers of the high dose groups in both sexes despite the upregulation of genes mediated by the heterodimer complex of PPARα and RXRα. The observed decrease in RXRα gene expression may be the result of a negative feedback loop to attenuate PPARα-mediated hepatic lipid metabolism via the let-7-RNF8-RXRα axis (Yagai et al., 2021).

PPARα helps to maintain systemic and cellular energy homeostasis by modulating the expression of genes involved in both peroxisomal and mitochondrial fatty acid β-oxidation (Aoyama et al., 1998). Hepatic transcriptomic responses to HFPO-DA exposure in mice consisted of induction of mitochondrial and peroxisomal activity at similarly low median BMDs (i.e., approximately 0.3 mg/kg HFPO-DA for enriched gene sets associated with mitochondrial and peroxisomal fatty acid metabolism). PPARα mediates the activity of these organelles through transcriptional coactivation with peroxisome proliferator-activated receptor-gamma coactivator 1-alpha (PGC1α). In addition to activity, the abundance of peroxisomes and mitochondria (i.e., biogenesis) is also co-regulated in a PPAR- and PGC1α-dependent manner (Fransen et al., 2017). Mitochondrial biogenesis gene sets were significantly enriched in livers from HFPO-DA-exposed mice, but at higher BMDs compared to fatty acid metabolism processes (i.e., median BMDs between 2–2.5 mg/kg HFPO-DA).

In addition to upregulation of PPARα signaling and fatty acid β-oxidation related gene sets, complement and coagulation cascades gene sets were highly significantly downregulated. The complement and coagulation cascades are part of the innate immune system and are made up of small proteins synthesized by the liver and secreted into the bloodstream (Janeway et al., 2001). Unlike the adaptive immune system, which develops immunological memory using antibodies after an initial exposure to a specific pathogen, the innate immune system uses phagocytes and inflammatory signaling (e.g., cytokines and chemokines) to resolve a pathological insult (Grabacka et al., 2021). PPARα has an important and specific function within the innate immune system and acts predominantly through suppression of various inflammatory reactions. Endogenous and pharmacological PPARα agonists have been investigated for their potential therapeutic applications in several chronic inflammatory disorders (e.g., rheumatoid arthritis). The critical immunomodulatory role of PPARα has been recently reviewed by Grabacka et al. (2021).

Overall, hepatic transcriptomic responses to HFPO-DA exposure in mice are consistent across sexes and studies and demonstrate activation of the PPARα signaling pathway, with increased peroxisomal and mitochondrial fatty acid β-oxidation at lower concentrations, and decreased complement cascades and altered cell cycle and apoptosis related pathways at higher concentrations. Thus, these data indicate that the reported liver effects in mice (i.e., hypertrophy, mitosis, and apoptosis) are mediated through rodent-specific PPARα signaling mechanisms that do not occur in humans.

## Data Availability

RNA sequencing data for all samples used in these analyses are publicly available at NCBI’s Gene Expression Omnibus (https://www.ncbi.nlm.nih.gov/geo/) (GEO series accession number GSE202302).

## Footnotes

1
https://www.teflon.com/en/industries-and-solutions/industries

2
https://www.nafion.com/en/industries

3 Note: This is one of the lowest RfD values in USEPA’s IRIS database.
