# Supplementary material for: Evaluation of Transcriptomic Responses in Livers of Mice Exposed to the Short-Chain PFAS Compound HFPO-DA
Source: Front Toxicol. 2022 Jun 27;4:937168. doi: 10.3389/ftox.2022.937168 (PMC9271854; doi:10.3389/ftox.2022.937168)
Supplement: Supplementary file 2 [file DataSheet2.DOCX]

**Supplementary File 1. Sequencing quality criteria.**

**Table 1.** Sequencing quality criteria across samples.

| **Quality Criterion** | **Mean** | **Standard Deviation** |
| --- | --- | --- |
| Sequencing Depth | 8,297,317.38 | 3,264,936.79 |
| Number of Sequenced Probes per Sample | 16,072.98 | 2,890.38 |

**Table 2.** Identification of samples that failed sequencing quality criteria. Samples with a sequencing depth or number of sequenced probes less than two standard deviations below the mean (i.e., lower bound) were excluded from the analyses. See **Table 1** for the mean and standard deviation of each sequencing quality criterion.

| **Sample Number** | **Dose (mg/kg)** | **Sex** | **Sequencing Depth** | | **Number of Sequenced Probes** | |
| --- | --- | --- | --- | --- | --- | --- |
|  |  |  | **Total** | **Above lower bound?** | **Total** | **Above lower bound?** |
| 1 | 0 | female | 9612688 | TRUE | 17029 | TRUE |
| 2 | 0 | female | 9719253 | TRUE | 17980 | TRUE |
| 3 | 0 | female | 10441369 | TRUE | 18281 | TRUE |
| 4 | 0 | female | 10232093 | TRUE | 18521 | TRUE |
| 5 | 0 | female | 11523373 | TRUE | 18143 | TRUE |
| 6 | 0.1 | female | 11322575 | TRUE | 18727 | TRUE |
| 7 | 0.1 | female | 11784913 | TRUE | 19832 | TRUE |
| 8 | 0.1 | female | 13878254 | TRUE | 19261 | TRUE |
| 9 | 0.1 | female | 10516636 | TRUE | 18693 | TRUE |
| 10 | 0.1 | female | 11287230 | TRUE | 18689 | TRUE |
| 11 | 0.5 | female | 214826 | FALSE | 6588 | FALSE |
| 12 | 0.5 | female | 10563730 | TRUE | 19463 | TRUE |
| 13 | 0.5 | female | 10204611 | TRUE | 17781 | TRUE |
| 14 | 0.5 | female | 10397824 | TRUE | 16984 | TRUE |
| 15 | 0.5 | female | 8931267 | TRUE | 17213 | TRUE |
| 16 | 5 | female | 7952605 | TRUE | 17152 | TRUE |
| 17 | 5 | female | 9982023 | TRUE | 17101 | TRUE |
| 18 | 5 | female | 10778193 | TRUE | 17781 | TRUE |
| 19 | 5 | female | 1700120 | FALSE | 11922 | TRUE |
| 20 | 5 | female | 11262409 | TRUE | 17223 | TRUE |
| 21 | 0 | male | 9365709 | TRUE | 16992 | TRUE |
| 22 | 0 | male | 9226740 | TRUE | 17917 | TRUE |
| 23 | 0 | male | 7780828 | TRUE | 14630 | TRUE |
| 24 | 0 | male | 8012441 | TRUE | 15728 | TRUE |
| 25 | 0 | male | 6947711 | TRUE | 16246 | TRUE |
| 26 | 0.1 | male | 9566505 | TRUE | 15508 | TRUE |
| 27 | 0.1 | male | 7704189 | TRUE | 15737 | TRUE |
| 28 | 0.1 | male | 8699739 | TRUE | 16808 | TRUE |
| 29 | 0.1 | male | 4012015 | TRUE | 13495 | TRUE |
| 30 | 0.1 | male | 10451739 | TRUE | 17843 | TRUE |
| 31 | 0.5 | male | 6681593 | TRUE | 15495 | TRUE |
| 32 | 0.5 | male | 2608954 | TRUE | 11347 | TRUE |
| 33 | 0.5 | male | 8661083 | TRUE | 16398 | TRUE |
| 34 | 0.5 | male | 10548222 | TRUE | 15832 | TRUE |
| 35 | 0.5 | male | 6943646 | TRUE | 15859 | TRUE |
| 36 | 5 | male | 4147445 | TRUE | 13091 | TRUE |
| 37 | 5 | male | 9098192 | TRUE | 14749 | TRUE |
| 38 | 5 | male | 2101495 | TRUE | 11446 | TRUE |
| 39 | 5 | male | 6088425 | TRUE | 14939 | TRUE |
| 40 | 5 | male | 940032 | FALSE | 8495 | FALSE |

**Table 3.** PCA analysis before and after removal of samples per a priori sequencing quality criteria.

| **Sample Number** | **Dose (mg/kg)** | **Sex** | **All Samples** | | **Samples Removed** | |
| --- | --- | --- | --- | --- | --- | --- |
|  |  |  | **PC1** | **PC2** | **PC1** | **PC2** |
| 1 | 0 | female | -17.532624 | 5.39200717 | -20.19531099 | 4.312049985 |
| 2 | 0 | female | -15.751298 | 6.60974281 | -18.67896874 | 5.487353392 |
| 3 | 0 | female | -10.487323 | 8.65381516 | -12.12104815 | 7.359843985 |
| 4 | 0 | female | -18.488806 | 5.73428212 | -21.40436756 | 5.015672562 |
| 5 | 0 | female | -18.120998 | 6.66605262 | -21.44009398 | 6.141013588 |
| 6 | 0.1 | female | -15.64461 | 5.30766934 | -18.11734168 | 3.99800698 |
| 7 | 0.1 | female | -15.163504 | 5.96208884 | -17.76205297 | 5.451905015 |
| 8 | 0.1 | female | -16.658729 | 6.35961078 | -18.71639275 | 5.486728629 |
| 9 | 0.1 | female | -17.981926 | 3.47661236 | -20.93451858 | 2.419984666 |
| 10 | 0.1 | female | -18.168338 | 4.63592423 | -20.9500732 | 3.778281986 |
| 11 | 0.5 | female | -30.599888 | -15.340226 | ⏤ | ⏤ |
| 12 | 0.5 | female | -16.104198 | 1.5799872 | -18.55710472 | 0.373411915 |
| 13 | 0.5 | female | -13.650771 | -0.9965945 | -15.90149401 | -3.036697261 |
| 14 | 0.5 | female | -11.585454 | 0.11512389 | -13.61287156 | -1.240196569 |
| 15 | 0.5 | female | -14.995734 | 1.180968 | -17.58134361 | -0.25063376 |
| 16 | 5 | female | -3.7931005 | -15.203529 | -5.324208431 | -19.58753217 |
| 17 | 5 | female | -2.6484808 | -15.221542 | -4.609950536 | -19.75513338 |
| 18 | 5 | female | -3.3340554 | -15.338687 | -5.251389337 | -20.03587913 |
| 19 | 5 | female | -2.951484 | -18.32173 | ⏤ | ⏤ |
| 20 | 5 | female | -8.1418722 | -12.503802 | -10.15582647 | -16.13511468 |
| 21 | 0 | male | 9.85988325 | 11.0225166 | 11.74212268 | 10.94226825 |
| 22 | 0 | male | 7.55890987 | 11.9655191 | 8.054590466 | 11.52783383 |
| 23 | 0 | male | 15.3578612 | 12.4271914 | 17.17922055 | 13.39845396 |
| 24 | 0 | male | 12.0939559 | 9.89172475 | 13.568164 | 9.995232185 |
| 25 | 0 | male | 10.6958067 | 10.9832114 | 12.11445246 | 11.98811019 |
| 26 | 0.1 | male | 15.1797387 | 10.7416024 | 16.7688639 | 10.55927482 |
| 27 | 0.1 | male | 13.9392283 | 12.2004233 | 16.2508718 | 12.22843783 |
| 28 | 0.1 | male | 14.2334374 | 7.53046293 | 15.6898219 | 6.848035403 |
| 29 | 0.1 | male | 13.6697149 | 10.0076651 | 15.62744815 | 10.65211881 |
| 30 | 0.1 | male | 12.059341 | 9.87552965 | 14.01475378 | 9.330928984 |
| 31 | 0.5 | male | 13.2358015 | 4.25559825 | 14.65474617 | 3.762044445 |
| 32 | 0.5 | male | 15.6527541 | 3.27291858 | 17.14542714 | 3.030291067 |
| 33 | 0.5 | male | 15.9328808 | 3.2048554 | 17.70711518 | 1.49032241 |
| 34 | 0.5 | male | 14.358175 | -2.4583225 | 16.02536431 | -4.396801492 |
| 35 | 0.5 | male | 14.0220583 | 3.38218081 | 15.80964984 | 3.421044518 |
| 36 | 5 | male | 15.9921796 | -14.273212 | 16.82202524 | -18.86198096 |
| 37 | 5 | male | 14.5270521 | -15.228692 | 14.97150318 | -19.79617373 |
| 38 | 5 | male | 13.1962453 | -19.208169 | 13.634429 | -25.02594917 |
| 39 | 5 | male | 13.1813258 | -15.926511 | 13.53378754 | -20.87655711 |
| 40 | 5 | male | 17.0568433 | -22.414267 | ⏤ | ⏤ |

**Figure 1.** Sequencing depth across samples. Samples with a sequencing depth two standard deviations below the mean sequencing depth of all samples, i.e., less than 1,767,443.8 (below the red line), were excluded from the analyses.

**Figure 2.** Number of sequenced probes per sample. Samples with a total number of sequenced probes less than two standard deviations below the mean number of probes sequenced per sample, i.e., less than 10,292.2 (below the red line), were excluded from the analyses.
